# Developmental fates and N_2_-fixing efficiency of terminally-differentiated versus undifferentiated bacteroids from legume nodules

**DOI:** 10.1093/plphys/kiaf613

**Published:** 2025-12-09

**Authors:** Carmen Sánchez-Cañizares, Raphael Ledermann, Joseph McKenna, Thomas J Underwood, Marcela Mendoza-Suárez, Rob Green, Karunakaran Ramakrishnan, Alison K East, Isabel Webb, Charlotte Kirchhelle, Beatriz Jorrín, Gerhard Saalbach, Euan K James, Flavia Moreira-Leite, Jason Terpolilli, Philip S Poole

**Affiliations:** Molecular Plant Sciences Section, Department of Biology, University of Oxford, Oxford OX1 3EL, UK; Molecular Plant Sciences Section, Department of Biology, University of Oxford, Oxford OX1 3EL, UK; School of Life Sciences, University of Warwick, Coventry CV4 7AL, UK; Molecular Plant Sciences Section, Department of Biology, University of Oxford, Oxford OX1 3EL, UK; Molecular Plant Sciences Section, Department of Biology, University of Oxford, Oxford OX1 3EL, UK; Department of Molecular Microbiology, John Innes Centre, Norwich NR4 7UH, UK; Department of Molecular Microbiology, John Innes Centre, Norwich NR4 7UH, UK; Molecular Plant Sciences Section, Department of Biology, University of Oxford, Oxford OX1 3EL, UK; Molecular Plant Sciences Section, Department of Biology, University of Oxford, Oxford OX1 3EL, UK; Molecular Plant Sciences Section, Department of Biology, University of Oxford, Oxford OX1 3EL, UK; Molecular Plant Sciences Section, Department of Biology, University of Oxford, Oxford OX1 3EL, UK; Proteomics Facility, Department of Biochemistry and Metabolism, John Innes Centre, Norwich NR4 7UH, UK; Department of Ecological Sciences, James Hutton Institute, Invergowrie, Dundee DD2 5DA, UK; Department of Biological and Medical Sciences, Oxford Brookes University, Oxford OX3 0BP, UK; Legume Rhizobium Sciences, Food Futures Institute, Murdoch University, 90 South St, Murdoch, Western Australia 6150, Australia; Molecular Plant Sciences Section, Department of Biology, University of Oxford, Oxford OX1 3EL, UK

## Abstract

Within legume root nodules, rhizobia differentiate into bacteroids, which reduce N_2_ into NH_3_ for secretion to the plant. Bacteroids may be swollen and terminally differentiated or non-swollen and can regenerate outside nodules. It is unclear why these different endosymbiotic lifestyles exist and whether they differ in symbiotic efficiency. Here, we compared N_2_ fixing bacteroids of the near isogenic strains *Rhizobium leguminosarum* bv. phaseoli 4292 (Rlp4292) and *R. leguminosarum* bv. viciae A34 (RlvA34), nodulating *Phaseolus vulgaris* (common bean) and *Pisum sativum* (pea), respectively. The larger bean plants fixed more N_2_, but peas fixed 1.6–3-fold more per unit nodule mass. Values per unit volume were similar between bean and pea because bean nodules are 2.7-fold denser (i.e. mass per unit volume). Bean nodules have higher numbers of smaller (∼1/5 the volume) bacteroids than peas. Bean bacteroids are denser (i.e. 2.5-fold protein per unit volume) although less closely packed than pea bacteroids (i.e. more space between bean bacteroids). Critically, pea bacteroids fix N_2_ at higher rates versus bean per unit bacteroid protein, as protein expression is skewed toward N_2_ fixation and TCA-cycle enzymes. Pea bacteroids infect 1.6 times the percentage of nodule volume of beans (i.e. 14.2% versus 9.1%). Overall, the increased packing density of pea bacteroids, as well as the bias of their proteome to nitrogenase, associated N_2_ fixation processes, and dicarboxylate metabolism, contributes to their greater symbiotic efficiency, which is likely driven by plant antimicrobial peptides.

## Teaser

The higher symbiotic efficiency of pea bacteroids arises from their greater packing density and proteomic bias toward nitrogenase and dicarboxylate metabolism.

## Introduction

Rhizobia are Alpha- or Beta-proteobacteria that reduce atmospheric N_2_ to NH_3_ in symbiosis with a wide variety of leguminous plants (González et al. 2019), with the bacteria accommodated inside root nodules. N_2_ fixation by legumes is a key component of the biosphere's biologically available nitrogen, with crop legume—rhizobia symbioses providing some 21 Tg annually of nitrogen, while forage and fodder legume—rhizobia symbioses contribute approximately 12 to 25 Tg (Herridge et al. 2008). Establishment of the symbiosis involves signal exchange, where the bacteria detect flavonoids released from legume roots via the receptor protein NodD, which is the transcriptional activator of a large group of *nod* genes. The encoded Nod proteins (e.g. NodOTNMLEFDABCIJ in the *Pisum sativum* or pea symbiont *Rhizobium leguminosarum* biovar *viciae*) synthesize short oligomers of four or five amino sugars, decorated with a long chain fatty acid, forming lipochitooligosaccharides (LCOs) (Spaink et al. 1991). LCOs are perceived by LysM receptors in the plant, which initiate the common symbiosis (Sym) pathway, the first part of which is activated in the plant by both nodulating rhizobia and mycorrhizal fungi (Gleason et al. 2006; Bozsoki et al. 2020). Although best known for nodulating legumes, rhizobia undergo a complex variation in life cycle from free-living bacteria in soil, to root-colonizing members of the microbiota through to infection of a legume host, typically via root hairs (Wheatley et al. 2020). The rhizobia grow down root hairs via an infection thread and are eventually engulfed by host cells and surrounded by a plant-derived symbiosome (also called peribacteroid) membrane (Poole et al. 2018). Plant cells become densely packed with bacteria, with their endosymbiotic N_2_-fixing form known as bacteroids.

Inside legume root nodules, bacteroids shut down expression of many genes, concentrating on expression of N_2_ fixation (*nif* and *fix*) genes (Karunakaran et al. 2009; Green et al. 2019) and secreting the ammonia they produce to the host, where it is incorporated into amino acids (Allaway et al. 2000). In return, bacteroids receive a carbon source in the form of C_4_ dicarboxylic acids (fumarate, succinate, and malate) to generate ATP and reductant for N_2_ fixation (Lodwig and Poole 2003). Modeling and ^13^C metabolic flux analysis indicate that O_2_ limitation restricts the decarboxylating arm of the TCA cycle in bacteroids, which limits ammonia assimilation into glutamate, forcing bacteroids to secrete this fixed N_2_ to the plant (Schulte et al. 2021). Consequently, bacteroids become symbiotic auxotrophs, dependent on the plant for branched-chain amino acids, even though they synthesize them as free-living bacteria (Lodwig et al. 2003; Prell et al. 2009).

Nodule morphology can vary between legume tribes, and based on differences in size, shape, and infection by rhizobia, several distinct nodule types have been described (Sprent et al. 2017). Two major groups include the determinate and indeterminate type nodules, which differ to each other in their morphology and development. Determinate nodules (e.g. *Phaseolus vulgaris* or common bean and *Glycine max* or soybean) are typically spherical, lack a persistent meristem (a region of actively dividing cells), and do not show a clear developmental gradient. Indeterminate nodules (e.g. pea and *Medicago truncatula*) have an elongated, branched, or lobed shape, and possess a persistent apical meristem, with the nodule divided into distinct zones which delineate different physiological states of both legume cells and rhizobia (meristematic, infection, N_2_ fixation, and senescence zones).

Inside root nodules at the bacteroid level, legumes of the Invert-Repeat Lacking Clade (IRLC), including pea and *M. truncatula*, can produce hundreds of nodule cysteine rich (NCR) antimicrobial peptides in root nodule cells, causing bacteroids to form multiple copies of their chromosome by endoreduplication. These polyploid endosymbiotic cells are terminally differentiated because they lose their ability to return to free-living growth and regenerate outside of the nodule, as they cannot undergo cell division with multiple copies of their chromosomes (Mergaert et al. 2006; Van de Velde et al. 2010; Wang et al. 2010). Terminally differentiated bacteroids are usually elongated or irregular in shape, forming Y-shaped cells with leaky membranes, that are swollen in size compared to free-living rhizobia, with symbiosomes within infected plant cells occupied by a single bacteroid. In contrast, non-IRLC legumes such as common bean and soybean do not produce NCR peptides, so bacteroids retain a chromosome number of 1 to 2, have a simple rod shape reminiscent in size to free-living cells, and retain the capacity to resume growth and regenerate outside the nodule, with multiple bacteroids occupying each symbiosome (Maróti and Kondorosi 2014; Poole et al. 2018).

While the evolutionary driver for the development of terminally differentiated and swollen bacteroids is not clear, it seems the attack of antimicrobial peptides in IRLC legumes reprograms bacteroids as ammonia secreting organelle-like organisms (also known as “ammoniaplasts”), which may increase the efficiency of N_2_ fixation (Oono and Denison 2010; Farkas et al. 2014; Penterman et al. 2014; Montiel et al. 2017; Sankari et al. 2022). In their study of pea (producing terminally differentiated swollen bacteroids) with common bean (nonswollen and undifferentiated bacteroids), Oono and Denison (2010) compared the symbiotic efficiency of both legumes inoculated separately with *R. leguminosarum* bv. *viciae* A34 (RlvA34). However, RlvA34 is a transgenic strain, produced by curing the common bean nodulating strain *R. leguminosarum* bv. *phaseoli* 4292 (Rlp4292) of its symbiotic (Sym) plasmid (pRP2) and replacing it with Sym plasmid pRL1J from pea-nodulating strain *R. leguminosarum* 248. RlvA34 therefore carries a symbiosis plasmid compatible for N_2_ fixation with peas, but not common bean (Hirsch 1979; Lamb et al. 1982), making measurements of symbiotic efficiency between pea and common bean inoculated with RlvA34 alone, difficult to compare. Therefore, to quantitatively compare efficiency between the distinct bacteroid types in these two legume hosts, we selected the strains Rlp4292 and RlvA34, that efficiently nodulate and fix N_2_ in common bean or pea nodules, respectively. The near-isogenic nature of these two strains, differing only in their Sym plasmid, but otherwise sharing a core genome, facilitates direct comparison of nodule and bacteroid N_2_ fixation efficiency between common bean and pea. Transcriptomic analysis of the determinate and indeterminate nodules formed by Rlp4292 and RlvA34, respectively, provides detailed bacteroid gene expression data (Green et al. 2019). We also sought to map the genetic, proteomic, and developmental changes from the whole plant to nodule and cellular levels to understand how these different developmental fates alter the symbiotic interaction between legumes and rhizobia and to provide a solid benchmark against which quantitative parameters that distinguish the two bacteroid types and their inherent efficiency could be compared.

## Results

### Modelling nodulation properties of bean and pea

To develop a coherent model of N_2_ fixation in which bacteroid types with different developmental fates are compared, we considered it essential to assess their efficiency at all physiological levels, starting at the whole plant level, then moving to the nodule level, and finishing at the cellular level. By using Rlp4292 and RlvA34 as our model strains, we enabled the closest comparison on nodulation and N_2_ fixation between terminally differentiated versus undifferentiated bacteroids, the two primary bacteroid types induced by rhizobia and associated in this study with determinate nodule-forming common bean *(P. vulgaris*) and indeterminate nodule-forming peas (*P. sativum*), respectively (Green et al. 2019). These strains constitute a unique resource because of their genetic similarity, differing only in a small region of their genome—their Sym plasmids. Their high degree of genetic homology provides the best possible comparative system to perform detailed physiological, biochemical, and microscopy analyses, that were not achieved in previously published studies to assess N_2_ fixation effectiveness. These two strains are effective relative to other characterized rhizobia that infect *P. vulgaris* and *P. sativum* (Supplementary Table S1) (Mwenda et al. 2018). Likewise, the hosts *P. vulgaris* cv. Tendergreen and *P. sativum* cv. Avola performed similarly relative to other tested cultivars (Supplementary Table S1) (Mwenda et al. 2018). Initial measurements of dry weight, nodule number, nodule mass, and nodule volume were made on plants at flowering, when nitrogenase activity is at a maximum, and hence a logical point to compare peak efficiency. Bean plants were larger than peas with a 1.9-fold higher shoot dry weight (Table 1). Reflecting their larger size, beans produced on average, a 1.4-fold increase in number of nodules. Although bean nodules were spherical in shape, compared to the cylindrically shaped pea nodules, their nodules were similar in volume to pea nodules (6.07 ± 0.48 versus 4.61 ± 0.22 mm^3^, respectively), so the higher nodule number was reflected in a similar 1.8-fold greater total nodule volume per bean plant. This led to an expectation of a similar increase in total nodule mass in beans relative to peas. However, the nodule dry biomass of beans was 4.7-fold greater than in peas, rather than the expected 1.4 to 1.8-fold. Thus, the biomass per nodule in beans is 3.4-fold greater than peas. Similarly, the biomass per unit volume is 2.7-fold higher in beans than peas. The startling conclusion is that the density of bean nodules is almost three times that of pea nodules.

**Table 1.** Comparison of *P. vulgaris* (bean) inoculated with Rlp4292 versus *P. sativum* (pea) inoculated with RlvA34

Parameter	Bean with Rlp4292	Pea with RlvA34	Fold change^a^	*P* value^b^
Plant and nodule comparison				
Plant shoot dry mass (mg/plant)	610 ± 50.2	321 ± 33.2	1.90	<0.001
Plant nodule fresh mass (mg nodules/plant)	1,445.3 ± 94.8	251.40 ± 13.35	5.82	<0.001
Plant nodule dry mass (mg nodules/plant)	81.8 ± 9.4	17.3 ± 1.85	4.72	<0.001
Nodule number (nodules/plant)	125 ± 10.3	88.6 ± 4.18	1.41	0.006
Nodule fresh mass (mg/nodule)	5.54 ± 0.70	2.56 ± 0.19	2.16	<0.001
Nodule dry mass (µg/nodule)	642 ± 25.1	195 ± 18.4	3.29	<0.001
Plant nodule volume (mm^3^ nodule/plant)	709 ± 43	400 ± 6.9	1.77	<0.001
Mean nodule volume (mm^3^/nodule)	6.07 ± 0.48	4.61 ± 0.22	1.32	0.013
Nodule density (mg/cm^3^ nodule)	115 ± 13.3	43.2 ± 4.64	2.67	<0.001
C_2_H_2_ Reduction per				
Plant (µmol/h/plant)	3.31 ± 0.39	1.92 ± 0.20	1.72	0.007
Plant (µmol/h/g SDW)	5.28 ± 0.25	6.00 ± 0.13	0.88	0.022
Nodule volume (µmol/h/cm^3^ nodule)	4.67 ± 0.56	4.81 ± 0.50	0.97	0.862
Nodule dry mass (nmol/h/µg nodule)	40.7 ± 2.60	124 ± 16.7	0.33	<0.001
Nodule infection and packing				
Calculated nodule volume (mm^3^/nodule)	5.40 ± 1.45	5.16 ± 0.59	1.05	0.881
Infected nodule volume (mm^3^/nodule)	1.36 ± 0.40	1.41 ± 0.23	0.97	0.917
Proportion infected nodule volume (%)	24.2 ± 1.52	26.2 ± 2.19	0.92	0.473
Bacteroid volume and surface area				
Volume (µm^3^/bacteroid)	0.93 ± 0.02	4.50 ± 0.09	0.21	<0.001
Surface area (µm^3^/bacteroid)	6.73 ± 0.14	21.1 ± 0.36	0.32	<0.001
Surface area: volume ratio	7.44 ± 0.06	4.78 ± 0.03	1.56	<0.001
Proportion bacteroids/infected cell (%)	36 ± 0.9	52 ± 2.3	0.69	<0.001
Total bacteroid volume (mm^3^/nodule)	0.49 ± 0.14	0.73 ± 0.12	0.67	0.235
Bacteroid protein				
Protein (pg/bacteroid)	0.32 ± 0.02	0.62 ± 0.03	0.52	<0.001
Number (bacteroids × 10^8^/nodule)	5.28 ± 1.56	1.63 ± 0.26	3.24	0.036
Protein density per bacteroid (pg/µm^3^ bacteroid)	0.34 ± 0.02	0.14 ± 0.01	2.42	<0.001
Protein density per nodule (µg/µm^3^ nodule)	340 ± 20.5	137 ± 6.53	2.48	<0.001
Free-living cells				
Volume (µm^3^/cell)	1.66 ± 0.10	1.77 ± 0.44	0.94	0.389
Surface area (µm^2^/cell)	8.34 ± 0.32	8.68 ± 0.26	0.96	0.420
Surface area: volume ratio	5.82 ± 0.13	5.10 ± 0.58	1.14	<0.001
^15^N_2_ Fixation rate per				
Plant (µmol/h/plant)	1.89 ± 0.22	0.58 ± 0.05	3.26	<0.001
Plant (µmol/h/g SDW)	1.33 ± 0.11	1.07 ± 0.12	1.24	0.143
Nodule dry mass (µmol/h/µg nodule)	9.76 ± 0.76	15.6 ± 1.26	0.63	0.001
Nodule volume (µmol/h/cm^3^ nodule)	1.22 ± 0.14	1.12 ± 0.09	1.09	0.552
Volume infected nodule (nmol/h/mm^3^)	5.15 ± 0.66	4.10 ± 0.30	1.26	0.147
Bacteroid (amol/h/bacteroid)	13.3 ± 1.70	35.4 ± 2.57	0.38	<0.001
Bacteroid protein (amol/h/pg protein)	42.2 ± 5.4	56.8 ± 4.1	0.74	0.043
Bacteroid volume (amol/h/µm^3^ bacteroid)	14.4 ± 1.83	7.87 ± 0.57	1.83	0.003

Plants harvested at flowering (35 d post inoculation for beans and 28 d post inoculation for pea) at peak nitrogenase activity. Values represent the average plus/minus standard error, with sample sizes per bean and pea treatment, respectively of: 11 each for plant and nodule comparison and C_2_H_2_ reduction; eight each for nodule infection and packing; 240 and 246 for bacteroid volume, surface area; six for bacteroid protein; 75 and 132 for free-living cells for bean and pea; nine and ten for ^15^N_2_ fixation rate for bean and pea.

^a^Fold-change determined as the ratio of the value for bean divided by pea.

^b^Student's unpaired t-Test, with significance set at 0.05.

Shoot Dry Weight abbreviated as SDW.

To measure nitrogenase activity, acetylene reduction was conducted on beans and peas at flowering (3.31 ± 0.39 versus 1.92 ± 0.20 *µ*moles C_2_H_2_ reduced/h/plant, respectively). The total acetylene reduction per plant was 1.7-fold greater in beans than peas (Table 1), which agrees very well with the 1.8-fold greater nodule volume of beans and the overall 1.9-fold greater biomass of bean plants compared to peas. Thus, after correction for volume, the rates were very similar in the beans versus peas (4.67 ± 0.56 versus 4.81 ± 0.50 *µ*moles C_2_H_2_ reduced/h/cm^3^ nodule, respectively). However, when the rates of acetylene reduction are calculated per unit mass of nodule, the rate in pea is 3-fold higher (124 ± 16.7 *µ*moles C_2_H_2_ reduced/h/µg nodules in pea versus 40.7 ± 2.60 *µ*moles C_2_H_2_ reduced/h/µg nodules in bean). As reported above, this is because the density of bean nodules per unit volume is 2.7-fold higher than in peas. If we consider that the nodule density and total mass per nodule reflects investment in resources, then pea nodules are more efficient at fixing N_2_ than beans. This raises fundamental questions about why bean nodules are much denser, which was next considered by dissecting the various components of the nodule, particularly bacteroid form, packing, and density (Table 1).

### Spatial packing of bacteroids within bean and pea nodules

**Figure 1.**
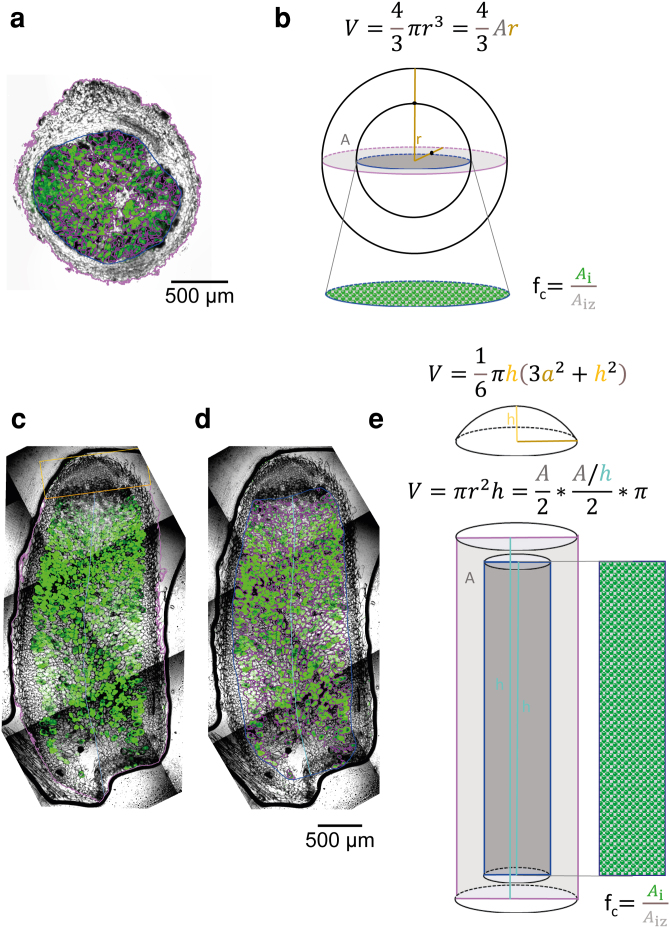
Volumes of bean and pea nodules. For spherical bean nodules **(A)**, whole nodule volumes were calculated **(B)** from the areas of cross sections (light purple outline). The volume of the infected zone (blue outline) was calculated analogously and the fractional colonization density (f_c_) was calculated as area (A_i_; represented by green autofluorescence of sfGFP-expressing bacteroids, outlined in dark purple in **A)** per infected zone (A_iz_). From these, the total infected volume (green) was calculated. For pea nodules **(C** and **D)**, each nodule was separated into an uninfected cap and a cylindrical body. Total nodule volumes were calculated **(E)** from the cap using its chord length and sagitta (orange) and the cylindrical body using its area (light purple) and length (teal). The volume of the infected zone (blue and teal) was calculated analogously. Fractional colonization density (f_c_) and final infected volume (green) was calculated as for bean nodules from the infected area (dark purple). See Materials and methods for details and formulae. Images in panels **C** and **D** are from the same composite image of a whole nodule with all tiles taken in a single continuous scan.

To establish the packing properties of bacteroids in bean and pea nodules, Rlp4292 and RlvA34 were fluorescently tagged with *sfGFP* expressed via a NifA-dependent promoter, to delineate the regions within nodules harboring N_2_-fixing bacteroids. Importantly, by using image analysis of sfGFP fluorescence, it was possible to separate the sizeable vacuoles present in pea cells from bacteroids. Longitudinal sections were then used to calculate the total nodule volume and the infected volume (i.e. the total volume of plant tissue colonized by N_2_-fixing bacteroids) (Fig. 1, Table 1). The average total volume from eight nodules was 5.40 ± 1.45 mm^3^ for beans and 5.16 ± 0.59 mm^3^ for peas, which is in good agreement with the volumes 6.07 ± 0.48 mm^3^ and 4.61 ± 0.22 mm^3^, determined by volume displacement of nodules (Table 1). The average volume of infected plant tissue was further calculated as 1.36 ± 0.40 mm^3^ or 24% of the total nodule volume for beans, and 1.41 ± 0.23 mm^3^ or 26% of the total nodule volume for peas, indicating that both types of nodules contain a similar volume of infected tissue despite their different morphology and organization. Having calculated the volume of a nodule occupied by infected plant tissue, the next step was to determine how much of the infected nodule volume was composed of bacteroids rather than plant cytoplasm. Our approach below was to use serial sectioning of nodules.

### Serial sectioning reveals the three-dimensional morphology and fine scale packing of bacteroids

**Figure 2.**
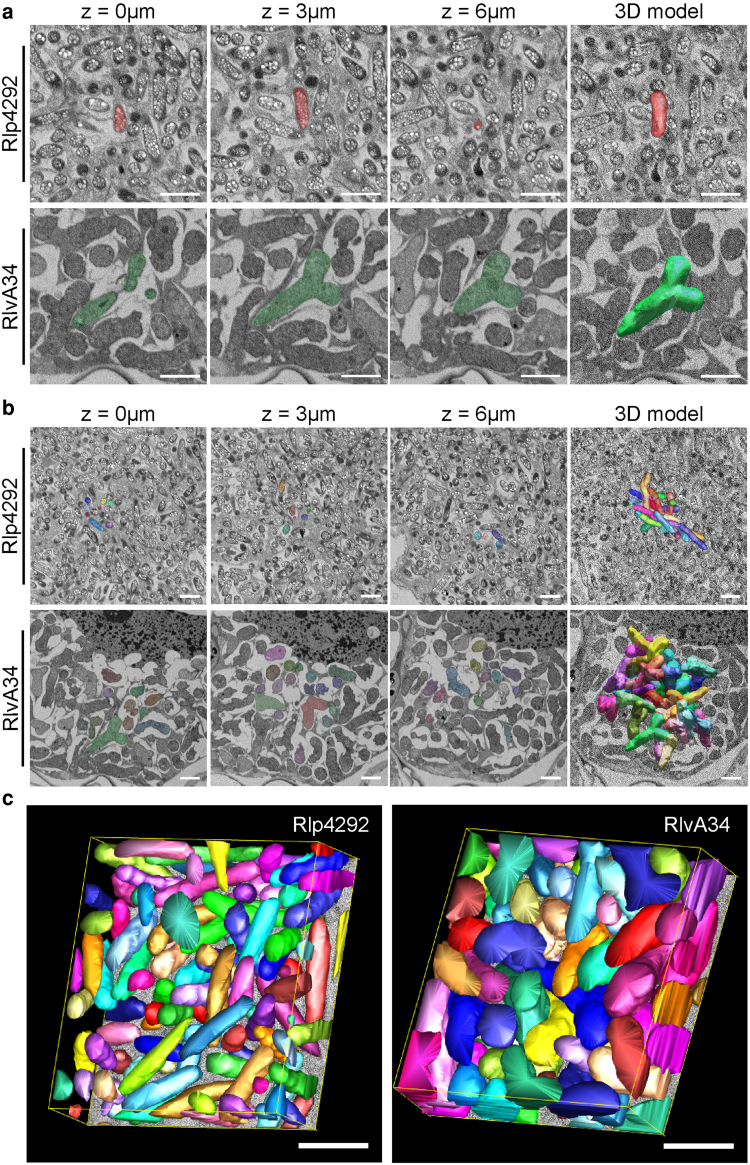
Volume electron microscopy imaging of individual bacteroids in pea and bean. **A)** SBF-SEM imaging of representative bacteroid in bean (RIp4292, red) and pea (RIvA34, green) segmented across example Z slices and their subsequent 3D reconstruction. **B)** Cluster of 30 bacteroids in bean (RIv4292) and pea (RIvA34) showing individual bacteroids segmented in different colors across a range of Z slices and 3D reconstruction of these bacteroids. **C)** Packing density of RIp4292 in bean, and RIvA34 in pea were determined by segmenting all bacteroid occupied volume in a defined cellular volume. All scale bars = 2 *μ*m.

To determine the three-dimensional (3D)-shape of bean and pea bacteroids in legume root nodules, we used Serial Block Face-Scanning Electron Microscopy (SBF-SEM), which has previously been used in plant cell biology to determine the organization of the plant vacuole during auxin induced growth repression (Scheuring et al. 2016), to confirm that protein storage vacuoles originate from a remodeled embryonic vacuole (Feeney et al. 2018), and to demonstrate the density of plasmodesmata (Yan et al. 2019). Sequentially captured Z-sections of osmium-labeled bean and pea nodule samples had sufficient contrast to allow SBF-SEM imaging and reconstruction of individual and clustered bacteroids (Fig. 2, Video 1), clearly showing the expected fundamental difference in bacteroid morphology between the samples with rod-shaped bean bacteroids, compared to Y-shaped pea bacteroids (Fig. 2A and B).

Further analysis and segmentation of the data allowed 3D volume and two-dimensional (2D) surface area determinations, showing that bean and pea bacteroids differ substantially in volume (0.93 ± 0.02 *µ*m^3^ in bean versus 4.50 ± 0.09 *µ*m^3^ in pea) and surface area (6.73 ± 0.14 *µ*m^2^ in bean versus 21.1 ± 0.36 *µ*m^2^ in pea). As would be expected, the smaller volume and surface area of bean bacteroids compared to pea bacteroids led to a greater surface area:volume ratio in bean compared to pea bacteroids (7.44 versus 4.78) (Table 1). To ensure there was no prior intrinsic difference in cell morphology between Rlp4292 and RlvA34 that could explain this variation in bacteroid size, we also measured volume and surface area of free-living cells grown on universal minimal salts UMS minimal media with confocal imaging (Table 1). There was no significant difference in either average cell surface area (8.34 ± 0.32 *μ*m^2^ for Rlp4292 and 8.68 ± 0.26 *μ*m^2^ for RlvA34 cells), or volume (1.66 ± 0.10 *μ*m^3^ for Rlp4292 and 1.77 ± 0.44 *μ*m^3^ for RlvA34) between the two strains. Therefore, pea bacteroid cells undergo significant enlargement inside pea nodules, differentiating into Y-shaped bacteroids with volumes almost five-fold greater than rod-shaped bean bacteroids.

While segmenting multiple individual bacteroids in the two datasets (Fig. 2), we observed that bean bacteroids appeared to be much less densely packed than bacteroids in pea (Fig. 2). This correlates with the observations made when imaging nodules with light microscopy (Fig. 1). To quantify the packing density of bacteroids, we segmented every bacteroid in a defined volume of the plant cell (Fig. 2C), allowing us to determine the ratio of plant cell volume occupied by bacteroids (Table 1). This showed that bean bacteroids occupy 36 ± 0.9% and pea bacteroids occupy 52 ± 2.3% of the volume of an infected cell. Applying the average volume of an infected plant cell determined above, a bean nodule has 0.49 ± 0.16 mm^3^, or 9.1% of the total nodule volume occupied by bacteroids, while the value for a pea nodule is almost 1.5-fold higher at 0.73 ± 0.15 mm^3^, or 14.2% of the total nodule volume occupied by bacteroids. However, as was shown above, bean bacteroids are almost five times smaller in volume than pea bacteroids. Taking these values into account allowed the calculation of the number of bacteroids per nodule for each nodule type. This showed that bean nodules are occupied by more than three times the number of bacteroids than pea nodules, with (5.28 ± 1.56) × 10^8^ bean bacteroids versus (1.63 ± 0.26) × 10^8^ pea bacteroids (Table 1). So, although bean and pea nodules are equivalent in volume, bean nodules carry a greater number of smaller bacteroids that are less closely packed within the nodule than in pea, a difference which is supported by the segmentation data in Fig. 2C.

### Nodule and bacteroid protein density

To obtain an accurate measurement of the protein content of a bacteroid, it was imperative to obtain a pure sample of bacteroids devoid of plant tissue. This was achieved by Percoll gradient centrifugation and protein measurement of purified bacteroids. At this stage, we realized that bean bacteroids are much denser than pea bacteroids, as they migrated further into Percoll gradients (Supplementary Figure S1). To normalize the protein contents, bacteroid counts from the same purified bacteroid samples were obtained by flow cytometry, using sYFP fluorescently tagged Rlp4292 and RlvA34 strains. This showed that bean bacteroids contained almost half the protein content of pea bacteroids (0.32 ± 0.02 pg protein/bacteroid versus 0.62 ± 0.03 pg protein/bacteroid). However, with almost 5-fold difference in volume between the two bacteroid types, the protein density within bean bacteroids is almost 2.5-fold higher than pea bacteroids (0.34 ± 0.02 versus 0.14 ± 0.01 pg bacteroid protein/µm^3^ bacteroid). Furthermore, as the volume of the infected zone containing bacteroids in bean nodules is smaller than that of pea nodules, the density of bacteroid protein per nodule is also almost 2.5-fold higher in beans than in peas (340 ± 20.5 versus 137 ± 6.53 *µ*g bacteroid protein/mm^3^ nodule, Table 1). Thus, relative to pea bacteroids, bean bacteroids have a higher protein density, and by extension, a greater protein density per infected nodule volume. The key question we next sought to address was whether these differences in bacteroid volume, packing and protein densities have any impact on the efficiency of symbiotic N_2_ fixation.

### N_2_ fixation at all scales

While acetylene reduction assay (ARA) provides an indirect estimate of nitrogenase activity by measuring ethylene production, ^15^N_2_ labeling methods offer a direct and more accurate quantification of N_2_ fixation by tracking the incorporation of labeled nitrogen into plant tissues. Due to its precision, the ^15^N_2_ approach is considered more reliable for estimating N_2_ fixation across different environmental and physiological conditions. Therefore, to compare N_2_ fixation rates between Rlp4292-inoculated beans and RlvA34-inoculated peas, ^15^N_2_ labeling assays were conducted on beans and peas at flowering time, as per the earlier ARA to assess nitrogenase activity. On a whole plant basis, the larger bean plants clearly fixed more N_2_ than the smaller pea plants, with a 3.26-fold higher ^15^N_2_ fixation rate (Table 1), consistent with the increased nitrogenase activity in beans measured earlier by ARA. Similarly, when expressed on a per nodule volume basis, this difference was nullified (1.22 ± 0.14 versus 1.12 ± 0.09 *µ*mol N_2_ fixed/h/cm^3^ nodule, Table 1), and the similar volumes of nodule tissue infected by bacteroids in both beans and peas also resulted in a nonsignificant difference in these rates (5.15 ± 0.66 versus 4.10 ± 0.30 nmol/h/mm^3^ infected nodule tissue). This indicates that bean and pea nodules are equivalent in symbiotic N_2_ efficiency. Bean nodules are occupied by a much greater number of bacteroids than pea nodules, so individual bean bacteroids on average fix 2.6-fold less N_2_ than RlvA34 bacteroids (13.3 ± 1.70 versus 35.4 ± 2.57 amol/h/bacteroid, Table 1) but when the large difference in volumes between these bacteroid types is considered, bean bacteroids fix almost twice as much N_2_ per unit bacteroid volume than pea bacteroids (14.4 ± 1.83 versus 7.87 ± 0.57 amol/h/µm^3^ bacteroid). Crucially though, the higher protein content of bean bacteroids mean they fix less N_2_ per unit protein than pea bacteroids (42.2 ± 5.4 versus 56.8 ± 4.1 amol/h/pg protein, *P* = 0.043). Indeed, when rates of N_2_ fixation are expressed per nodule dry weight (µmol/h/µg nodule), bean nodules are 1.6 times significantly less efficient at fixing N_2_ than pea nodules (9.76 ± 0.76 versus 15.6 ± 1.26, *P* = 0.001). Therefore, because protein density per bacteroid and per nodule is higher in bean nodules, pea bacteroids are more efficient at N_2_ fixation per unit protein, suggesting a fundamental difference in protein expression under N_2_-fixing conditions between the two bacteroid types. We therefore sort to explore the cause of this difference at the protein level.

### Mapping the proteome

**Figure 3.**
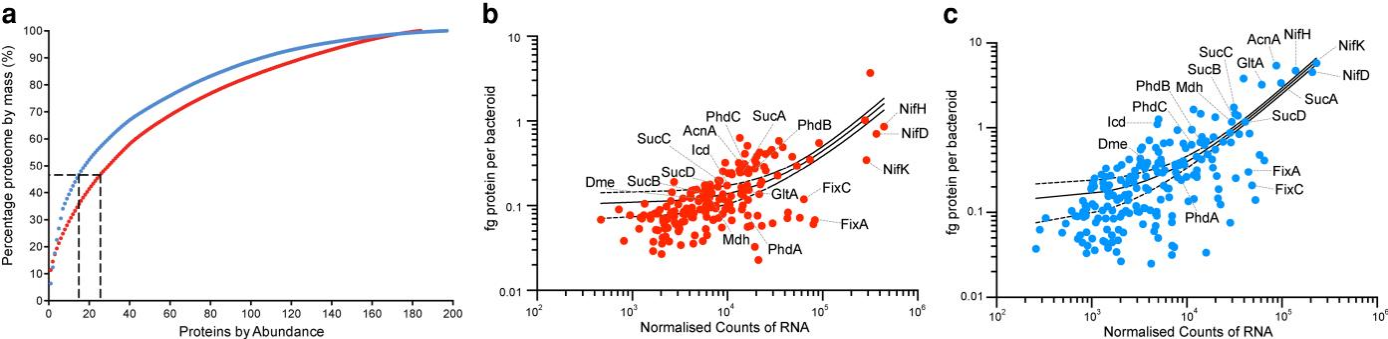
Relative protein and RNA abundance. **A)** Comparison of the overall abundance of proteins detected from quantitative proteomics of Rlp4292 bean (red dots) and RlvA34 pea (blue dots) bacteroids, expressed as a percentage of the mass of the proteome. Dotted line represents number of proteins constituting 50% of the proteome. **B)** Correlation between normalized counts of RNA (x-axis) and fg of protein per bean bacteroid (red dots). **C)** Correlation between normalized counts of RNA (x-axis) and fg of protein per pea bacteroid (blue dots). RNA data used to construct panel **B** and **C** from Green et al. (2019). Abbreviations for selected proteins in panels B and C: AcnA, Aconitate hydratase I; Dme, NAD^+^-dependent malic enzyme; FixA, Electron transfer flavoprotein, beta subunit; FixC, Electron transfer flavoprotein oxidoreductase; GltA, Citrate synthase I; Icd, Isocitrate dehydrogenase; Mdh, Malate dehydrogenase; NifD, Nitrogenase molybdenum-iron protein alpha chain; NifH, Nitrogenase iron protein; NifK, Nitrogenase molybdenum-iron protein beta chain; PdhA, Pyruvate dehydrogenase (PDH) E1 component alpha subunit; PdhB, PDH E1 component, beta subunit; PdhC, PDH E2 component dihydrolipoamide acetyltransferase; SucA, 2-oxoglutarate dehydrogenase (OGDH) E1 component; SucB, 2-OGDH E2 component; SucC, Succinyl-CoA synthetase, beta subunit; SucD, Succinyl-CoA synthetase, alpha subunit.

Quantitative proteomics was employed to compare protein abundance in bean and pea bacteroids, with a total of 184 and 197 proteins detected in bean and pea bacteroids, respectively. Comparison of the overall abundance between bacteroid types as a percentage of the mass of the proteome (Fig. 3A) showed a significant difference between protein populations in bacteroid types (*P* = 0.016, Komolgrov-Smirnov test). Pea bacteroids expressed their most abundant proteins to a much higher level, with 50% of the proteome made up of the top 18 proteins detected, in comparison to bean, where 30 proteins made up the first 50% of the proteome.

Bacteroid N_2_ fixation is dominated by C_4_-dicarboxylate metabolism, which bacteroids receive as a carbon source from the host plant. At the individual cell level, pea bacteroids showed greater abundance in dicarboxylate metabolizing enzymes NAD^+^-dependent malic enzyme (Dme, 3.24-fold), malate dehydrogenase (Mdh, 9.44-fold), and phosphoenolpyruvate carboxykinase (PckA, 1.42-fold) enzymes compared to bean (Table 2). Similarly, pyruvate dehydrogenase (PdhABC) and the enzymes of the decarboxylating arm of the TCA cycle (citrate synthase, GltA; aconitase, AcnA; isocitrate dehydrogenase; Icd, 2-oxoglutarate dehydrogenase; SucAB; succinyl CoA synthetase, SucCD), which are known to be highly active in N_2_-fixing bacteroids, were all increased in abundance in pea bacteroids relative to bean. Crucially, a much greater number of proteins constituting the nitrogenase enzyme complex (Fe protein NifH and Mo-Fe proteins NifD and NifK) were present in pea bacteroids, as well as a greater than 2.6-fold increase in essential electron transfer proteins FixA and FixC compared to Rlp4292, and alpha and beta subunits of the ATP synthase (Table 2). Therefore, pea bacteroids support higher rates of N_2_ fixation, as they express a greater abundance of nitrogenase enzyme and supporting proteins, on an individual cell basis, compared to bean bacteroids. Expressing the absolute protein abundance values per unit bacteroid volume, dramatically nullifies differences between pea and bean bacteroids, with the numbers of proteins (including NifH, NifD, and FixA) very similar, while others such as Dme, FixC, and PdhABC, are now more abundant in bean bacteroids. This change reflects the very large difference (4.5-fold) in volume between pea and bean bacteroids. Crucially, when these proteins are expressed per unit bacteroid protein, the abundance of NifHDK and TCA cycle enzymes is significantly higher in pea relative to bean bacteroids (Table 2). Thus, the pea bacteroid proteome, relative to that of bean, is biased toward production of nitrogenase and supporting enzymes. This may be a key outcome of endoreduplication of pea bacteroids by host plant NCR peptides. Absolute levels of individual proteins in bacteroids were also compared to their transcript levels measured by RNA-Seq (Green et al. 2019). For bean bacteroids, normalized RNA counts show a moderate positive correlation with mass of protein per bacteroid, with a Pearson correlation coefficient of r = 0.66 and R^2^ = 0.44 (Fig. 3B). In contrast, for pea bacteroids, the correlation is stronger, with r = 0.84 and R^2^ = 0.71 (Fig. 3C). Thus, there is a reasonable correlation between transcription and protein abundance in both types of bacteroids.

**Table 2:** Selected bacteroid proteins and their fold change relative to Rlp4292 (i.e. A34/4292)

			Fold change* (RlvA34/Rlp4292) per:				
Locus tag	Protein	Product	Bacteroid	Bacteroid volume	Nodule	Nodule volume	Bacteroid protein
Nitrogenase, electron transfer and ATP							
RHLv8088^a^	NifH	Nitrogenase iron protein	**5**.**49**	**1**.**13**	**1**.**63**	**2**.**15**	**2**.**78**
RHLv8087^a^	NifD	Nitrogenase molybdenum-iron protein alpha chain	**6.42**	**1.33**	**1.92**	**2.53**	**3.25**
RHLv8086^a^	NifK	Nitrogenase molybdenum-iron protein beta chain	**16.75**	**3.46**	**5.18**	**6.83**	**8.48**
RHLv8128^a^	FixA	Electron transfer flavoprotein, beta subunit	**4.99**	**1.03**	**1.47**	**1.93**	**2.53**
RHLv8126^a^	FixC	Electron transfer flavoprotein oxidoreductase	**2.64**	**0.55**	**0.82**	*1.08*	**1.34**
RHL3232	AtpA	Proton translocating ATP synthase, F1 alpha subunit	**3.88**	**0.80**	**1.16**	**1.52**	**1.96**
RHL3230	AtpD	ATP synthase, F1 beta subunit	**3.28**	**0.68**	*0.98*	**1.29**	**1.66**
RHL2934	-	Putative GTPases (G3E family)	**4.56**	*0.94*	**1.34**	**1.77**	**2.31**
Dicarboxylates and TCA cycle							
RHL1472	Dme	Malic enzyme, NAD^+^-dependent	**3.24**	**0.67**	*0.96*	**1.26**	**1.64**
RHL3264	Mdh	Malate dehydrogenase, NAD^+^-dependent	**9.44**	**1.95**	**2.82**	**3.71**	**4.78**
RHL3615	PckA	Phosphoenolpyruvate carboxykinase	**1.43**	**0.29**	**0.42**	**0.56**	**0.72**
RHL1082	PdhA	PDH E1 component, alpha subunit	**4.05**	**0.84**	**1.19**	**1.57**	**2.05**
RHL1083	PdhB	PDH E1 component, beta subunit	**2.48**	**0.51**	**0.74**	*0.97*	*1.25*
RHL1084	PdhC	PDH E2 component, dihydrolipoamide acetyltransferase	**2.03**	**0.42**	**0.61**	**0.80**	*1.03*
RHL1087	LpdA	PDH E3 component^b^, dihydrolipoamide dehydrogenase	**3.46**	**0.71**	*1.05*	**1.39**	**1.75**
RHL1075	GltA	Citrate synthase I	**21.19**	**4.38**	**6.32**	**8.32**	**10.73**
RHL3358	AcnA	Aconitate hydratase I	**16.86**	**3.48**	**5.03**	**6.62**	**8.54**
RHL1435	Icd	Isocitrate dehydrogenase, NADP-dependent	**6.42**	**1.33**	**1.92**	**2.52**	**3.25**
RHL3260	SucA	2-OGDH E1 component	**13.39**	**2.77**	**3.99**	**5.25**	**6.78**
RHL3258	SucB	2-ODDH E2 component	**10.28**	**2.12**	**3.13**	**4.12**	**5.21**
RHL3263	SucC	Succinyl-CoA synthetase, beta subunit	**8.93**	**1.85**	**2.67**	**3.51**	**4.52**

Abbreviations: 2-OGDH, 2-oxoglutarate dehydrogenase; PDH, pyruvate dehydrogenase.

^a^Locus tag for genes encoded on plasmid pRL1 in RlvA34. Corresponding genes on pRP2 in Rlp4292 are: *nifH* (RHLp7062 and RHLp7131), *nifD* (RHLp7061), *nifD* (RHLp7060), *fixA* (RHLp7092), and *fixC* (RHLp7094).

^b^Also, the E3 Component of 2-OGDH.

^*^Values in bold are significantly different (*P* < 0.05) according to Welch's t-Test, while those in italics are not.

## Discussion

In this work, we address the question of why legumes have evolved to produce terminally differentiated bacteroids. Previous studies had already shown that terminal differentiation enhances N_2_ fixation efficiency (Oono and Denison 2010; Kazmierczak et al. 2017; Montiel et al. 2017; Lamouche et al. 2019; Chen Wen et al. 2023). However, these earlier investigations had some significant limitations, including the use of suboptimal bacterial strains, nonphysiological growth conditions, or comparisons across plant species with inherently different physiologies. Therefore, we revisit this evolutionary question with an optimized comparative system using two extremely close strains of rhizobia that nodulate bean and pea, supported by comprehensive physiological, biochemical, and microscopic analyses not previously applied in this context. This comparative study highlights how the differentiation of rhizobia into bacteroids in root nodules leads to substantial changes in their physiology and metabolism, altering their symbiotic efficiency. Overall, we show that pea bacteroids relative to bean fix more N_2_ on an individual basis. This is accompanied by a much larger bacteroid size, which partly explains their increased N_2_ fixation. The nitrogenase complex and supporting enzymes are also more abundant in pea bacteroids (normalized by protein), with the proteome biased toward these proteins. This was also observed in a comparative study between pea (*P. sativum* cv. Frisson) and lentil (*Lens culinaris* cv. Magda) nodules from plants inoculated with RlvUPM791 (Durán et al. 2021), thus confirming the specialization of these endosymbiotic cells for the conversion of atmospheric nitrogen into ammonia. From transcriptomic analysis of pSym encoded genes from Rlp4292 bean and RlvA34 pea bacteroids, there is stronger upregulation of a smaller number of genes on pRL1JI (pea) than on pRP2 (bean) (Green et al. 2019). While the *nif and fix* gene clusters are >10-fold upregulated in both, in pea almost every other pRL1JI gene is downregulated >2-fold (and some >10-fold). This is not the case for beans where expression of most pRP2 genes is unaffected, with only a very small proportion downregulated >2-fold. In comparing the whole genomes, a similar picture emerges, more genes are downregulated and fewer genes are upregulated in pea than in bean bacteroids (Green et al. 2019). This transcriptomic data is in good agreement with the proteomic data presented in this study; in RlvA34 fewer genes are upregulated and more genes are downregulated which would lead to a lower number of different proteins in pea bacteroids.

Pea bacteroids are more densely packed within infected plant cells than bean bacteroids, although bean nodules harbor three-times more bacteroids than pea nodules. Bean nodules are 2.7-fold the density of pea nodules, partly because bean bacteroids are much denser than pea bacteroids. However, as bean and pea bacteroids respectively only make up 9.1% and 14.2% of the nodule volume, much of the increased density of bean nodules must be due to the inherent density of plant tissue. The striking change in pea nodules is not only the much larger cell volume of individual bacteroids but also that the packing and distribution of bacteroids leads to a 1.6-fold greater volume of infected nodule volume (i.e. 14.2% versus 9.1%). When the greater density of bean nodules is combined with the bias of the pea bacteroid proteome to nitrogenase and associated N_2_ fixation proteins, as well as dicarboxylate metabolism, the greater efficiency of pea versus bean nodules can be explained.

Based on a substantially robust and integrated dataset, our findings align with earlier conclusions. For example, in a characterization of symbiotic root nodules performed with *Bradyrhizobium arachidis* strain CCBAU051107, a strain which can form both determinate dalbergoid-type nodules on peanut (*Arachis hypogaea*) differentiating into swollen bacteroids, and indeterminate nodules on the woody shrub *Sophora flavescens* (nonswollen bacteroids), the authors observed the N_2_ fixation activity per unit mass of peanut nodules was 3-fold higher than that of S*. flavescens* (12.06 ± 2.04 versus 3.81 ± 0.58 nmol/h/mg, respectively) (Chen Wen et al. 2023). The expression levels of N_2_-fixing genes measured by RNA-Seq, were 2- to 5-fold greater in bacteroids of peanut nodules than those of *S. flavescens,* suggesting that differentiated bacteroids are more efficient (Chen Wen et al. 2023). A caveat is that this comparison is between nodules on an annual crop (peanut) with those on a woody shrub (*S. flavescens*), which are plants with radically different physiologies. Although similar conclusions for increased symbiotic efficiency were also reached comparing peas and common bean in the study by Oono and Denison (2010), RlvA34 was chosen as the only infecting strain for both pea and bean (Oono and Denison 2010). This is highly unusual as the RlvA34 Sym plasmid (pRLJI1) is specific for peas and lacks the specific *nod* genes that would allow bean nodulation. In the same study, Oono and Denison (2010) also compared the symbiotic efficiency of strain *Bradyrhizobium* sp. 32H1 inoculated on peanut (*A. hypogaea*) and cowpea (*Vigna unguiculata*), both symbiotic hosts forming determinate nodules, but hosting swollen and terminally differentiated versus nonswollen and undifferentiated bacteroids, respectively. The bacteroids of peanut nodules conferred more net host benefit in terms of nodule resource investment (plant growth per gram of nodule growth) and efficiency of N_2_ fixation (Oono and Denison 2010). However, while strain 32H1 is effective at fixing N_2_ with peanut, it is not highly effective on cowpea (Sen and Weaver 1981). Nevertheless, while some aspects of these previous studies have been limited by slightly indirect comparisons, they are suggestive that differentiated bacteroids are more efficient at N_2_ fixation than undifferentiated bacteroids.

The use in this work of near isogenic strains of *R. leguminosarum* (Rlp4292 and RlvA34), both of which are fully effective on their respective hosts of bean and pea (Supplementary Table S1; Mwenda et al. 2018), confers confidence that we are measuring real differences in efficiency, related to fundamentally different developmental fates of bacteroids. They broadly agree with studies on other legumes (Oono and Denison 2010; Chen Wen et al. 2023), and we have no reason to think that these quantitative measurements and differences would not be applicable to a wide variety of symbioses.

It is known that the endoreduplication and variations in ploidy level of pea bacteroids by NCR-like peptides encoded within the plant genome leads to enlarged bacteroids (Montiel et al. 2017). This difference in efficiency is linked to variations in ploidy level, with highly polyploid (containing multiple sets of chromosomes) spherical bacteroids being more efficient than the elongated ones with lower ploidy levels. Comparisons between terminally differentiated bacteroids with varying genome contents (6C in *Pisum* and 18C in *Vicia*, as reported by Mergaert et al. 2006) raises the question of whether higher ploidy levels correlate with enhanced metabolic capacity or symbiotic performance. These ploidy differences suggest that host-specific modulation of bacteroid differentiation could influence symbiotic efficiency and may represent an adaptive trait shaped by co-evolution. Indeed, studies of the determinate nodules of *Aeschynomene* demonstrate that plant host NCR peptides can induce formation of spherical bacteroids, which are associated with higher N_2_ fixation efficiency compared to elongated bacteroids, even though both are terminally differentiated (Lamouche et al. 2019). A protein atlas of *M. truncatula* contained entries for 252 detected and quantified NCR peptides showed their expression patterns changed during the stages of nodule development (Marx et al. 2016). NCR peptides seem to interfere with the bacterial cell cycle regulatory network, with bacteroids displaying a major drop in expression of the CtrA master regulator of the cell cycle and of its cognate regulon (Penterman et al. 2014; Pini et al. 2015). Addition of *Medicago truncatula* NCR peptide NCR247 to *S. meliloti* cultures slowed down or fully inhibited protein synthesis in a concentration-dependent manner (Tiricz et al. 2013). In bacteroids, protein synthesis continues, although the complexity of the proteome is reduced; with a large portion of proteins of <30 kDa reduced and others like nitrogenase and GroEL chaperone produced in high quantities (Farkas et al. 2014). In considering different legume species and their microsymbionts, such as *M. truncatula* and *Sinorhizobium* strains, there is often large strain variation in bacterial ploidy and efficiency of N_2_-fixation (Kazmierczak et al. 2017). In our work, we sort to avoid these differences by choosing highly efficient pairs of legume host and microsymbiont, but of course compatibility between a rhizobial strain and a legume is a major determinant of N_2_-fixation efficiency.

In RNA-Seq and microarray experiments examining bacteroids at 28 dpi (pea) or 35 dpi (bean), there is general downregulation of translation compared to free-living bacteria (Karunakaran et al. 2009; Green et al. 2019). However, there is specific downregulation of genes associated with cell division only in peas, consistent with endoreduplication (without cell division), a consequence of NCR peptide exposure (Green et al. 2019).

Overall, this work provides insights into the functional physiology of different bacteroid types and offers compelling evidence for the adaptive advantage conferred by terminal differentiation. Terminal bacteroid differentiation seems to be a plant-driven strategy to control the endosymbiont. By understanding why NCR peptide-producing legumes program bacteroids for greater N_2_-fixing efficiency, we can better inform efforts to improve symbiotic performance in crop species, with significant implications for sustainable agriculture and reduced reliance on synthetic fertilizers.

## Materials and methods

### Bacterial strains and growth conditions

*Escherichia coli* was grown in LB (Sambrook et al. 1989) at 37 °C with antibiotics added at the following concentrations if appropriate: Gentamicin 10 *µ*g/ml, Streptomycin 50 *µ*g/ml, Spectinomycin 50 *µ*g/ml, Ampicillin 100 *µ*g/ml. For *E. coli* ST18, 50 *µ*g/ml of δ-aminolevullinic acid was supplemented (Thoma and Schobert 2009) *R. leguminosarum* strains (Table 3) were grown at 28 °C in TY medium (Johnston and Beringer 1975) or UMS minimal medium (Terpolilli et al. 2016) with appropriate carbon and nitrogen sources at 10 mm unless otherwise stated. Antibiotics were added at the following concentrations where needed: Rifampicin 50 *µ*g/ml, Streptomycin 500 *µ*g/ml, Gentamicin 20 *µ*g/ml.

**Table 3:** Strains and plasmids used in this study

Strain or Plasmid	Relevant genotype or phenotype	Reference
*E. coli*		
DH5α	*supE44* Δ*lacU169* (ϕ80 *lacZ*ΔM15) *hsdR17 recA1 gyrA96 thi-1 relA2*	BRL, Gaithersburg, USA
ST18	Sm^R^*, pro thi hsdR*^+^; chromosome::RP4-2 Tc::Mu-Kan::Tn*7*/λpir Δ*hemA*	(Thoma and Schobert 2009)
*R. leguminosarum*		
4292	Rf^R^, biovar *phaseoli* wild type	(Lamb et al. 1982)
A34	Sm^R^, biovar *viciae* wild type	(Downie et al. 1983)
OPS1875	Sm^R^ Gm^R^, (A34) Tn7(P_J123104_-*sYFP*)	This work
OPS1877	Rf^R^ Gm^R^, (4292) Tn7(P_J123104_-*sYFP*)	This work
OPS2220	Sm^R^ Gm^R^, (A34) pOPS0875	This work
OPS2221	Rf^R^ Gm^R^, (4292) pOPS0875	This work
OPS3405	Sm^R^ Gm^R^, (A34) Tn*7*(P_J23104_-*mCherry*; P_s*nifH*_-*sfGFP*; *aacC1*)	This work
OPS3411	Rf^R^ Gm^R^, (4292) Tn*7*(P_J23104_-*mCherry*; P_s*nifH*_-*sfGFP*; *aacC1*)	This work
Plasmids		
pTNS3	Ap^R^, transposase delivery plasmid for integration of mini-Tn*7* cassettes	(Choi et al. 2008)
pOGG037	Sp^R^, pL0M-SC-*sfGFP*	This work
pOGG043	Sp^R^, pL0M-PU-P_s*nifH*_ (synthetic P*_nifH_*)	(Mendoza-Suárez et al. 2020)
pOGG157	Sp^R^, pL0M-T-*DT16*	(Geddes et al. 2019)
pOPS0696	P_J23104_-RBstd-*YFP-DT16*-pOGG024	This work
pOPS0875	Gm^R^, pneo-mCherry-pOGG024	This work
pUC18R6KminiTn*7*T	Ap^R^ Gm^R^, pUC18R6KminiTn*7*T	(Choi et al. 2008)
pOPS1015	Ap^R^ Gm^R^, pUC18T-miniTn7T-Gm-T1-P_J123104_-RBstd-*mCherry*-*DT16*-*T0*	(Westhoek et al. 2021)
pOPS1016	pUC18T-miniTn7T-Gm-T1-P_J123104_-RBstd-*sYFP-DT16-T0*	This work
pOPS1699	Ap^R^ Gm^R^, pUC18R6K-miniTn7T-Gm-T1-P_s*nifH*_-*sfGFP*-*DT16*	This work
pOPS2031	Ap^R^ Gm^R^, (pOPS1699) P_J23104_-*mCherry*, P_s*nifH*_-*sfGFP*-*DT16*	This work

### Plasmid and strain construction

To tag both RlvA34 and Rlp4292 wild type strains with a constitutive *mCherry* and a NifA-dependent *sfGFP*, we first assembled pOPS1699 via Golden Gate assembly from the following plasmids: pOGG037, pOGG043, pOGG157, and pUC18R6K-miniTn*7*T. This plasmid contains a mTn*7* module with *sfGFP* expressed by a synthetic *nifH* promoter (P_s*nifH*_). Subsequently, pOPS1699 was linearized with *Nsi*I and a fragment containing P_J23104_-*mCherry*, previously amplified from pOPS1015 using primers oxp5931 (AGCTAATTCGAGATCATGCAAGCTTCTCGAGGAATTCC) and oxp5932 (TCCGAATTCGGCATTATGCAATGAGCTCACTAGTGCTC), was inserted via HiFi assembly (New England Biolabs), so that is expressed in opposite direction to P_s*nifH*_-*sfGFP*. The resulting plasmid pOPS2031 was mobilized into both recipient strains in triparental matings using pTNS3 as a helper plasmid for mTn*7* transposition. For confocal imaging of free-living cells, we built plasmid pOPS0875 using Golden Gate cloning to express the *mCherry* module under the constitutive P_neo_ promoter in the medium-copy, broad-host range pOGG024 BEVA vector (Geddes et al. 2019). This plasmid was introduced into RlvA34 and Rlp4292 by conjugation. To tag both RlvA34 and Rlp4292 wild type strains with a constitutive sYFP for bacteroid protein measurements and flow cytometry analyses, we generated the vector pOPS1016. sYFP expression cassette was amplified from from the plasmid pOPS0696 with primers oxp1842 (CATGCATGAGCTCACTAGTGCTCTAGGGCGGCGG) and oxp1843 (CTTCTCGAGGAATTCCTGCAGCTGGATTCTCACCAATAAAAAACG), and cloned into pUC18T-miniTn7T-Gm, digested with BamHI and PstI, using BD In-Fusion^TM^ cloning kit (Clontech). This plasmid was introduced into RlvA34 and Rlp4292 by conjugation.

### Plant inoculation and growth

*P. sativum* cv. Avola and *P. vulgaris* cv. Tendergreen seeds were sterilized in 70% ethanol and 2% sodium hypochlorite followed by extensive washing in distilled water. Seeds were pregerminated on 0.8% w/v agar plates at 28 °C in the dark for two days. Seeds were then placed into 1 l beakers filled with washed medium grade vermiculite for peas, and 2 l beakers with fine grade vermiculite for beans. For peas, 400 ml of N-free rooting solution (Poole et al. 1994) was added to each pot prior to autoclaving and for beans, 400 ml of Broughton and Dilworth's nutrient solution (1971). Strains for inoculation were grown on TY slopes with appropriate antibiotics added. Cells were then resuspended in UMS and washed once and OD_600_ was adjusted to 0.01, corresponding to 1×10^7^ cfu/ml. A 1 ml suspension was used to inoculate each pot, with one plant per pot, and 11 plants per inoculated treatment. Plants were grown for 28 (*P. sativum*) or 35 (*P. vulgaris*) d at 21 °C with a 16 h photoperiod. *P. vulgaris* was watered weekly with 100 ml of sterile Broughton and Dilworth's nutrient solution.

### Acetylene reduction and ^15^N_2_ assays

Acetylene reduction was determined on plants incubated in 95% air: 5% acetylene for 1 h in 250 ml Schott bottles, as previously described (Allaway et al. 2000). Nodules were then excised, counted, and weighed fresh. Finally, root and shoot were separated, dried at 70 °C in a dry-heat incubator for three days then weighed. For ^15^N_2_ fixation, assays were conducted as previously published (Lodwig et al. 2003). Briefly, plants were incubated in 80% air: 20% ^15^N_2_ gas (98%+, Cambridge Isotope Laboratories, Inc. USA) for 2 h in 250 ml Schott bottles. The incorporation of ^15^N_2_ was determined separately for roots, shoots, and nodules. Tissue samples were then dried overnight at 70 °C, and dry weights were determined for each fraction separately. For each sample, 4 mg of dried tissue was then crushed and sent for combustion and mass spectrometric analysis. Total nitrogen and ^15^N_2_ enrichment were measured in a continuous flow isotope ratio mass spectrometer (James Hutton Institute, UK). From the δ^15^N (‰) values obtained separately for each plant fraction, biological nitrogen fixation (BNF) enrichment or δ atom percent excess (APE) was calculated for each sample by subtracting the ^15^N natural abundance (0.36782) from the amount of atmospheric N_2_ fixed by each plant fraction (^15^N Atom %). Then, the N_2_ fixed/h for each fraction was calculated as ((δ APE)/100)*total N*5/4), to account for the 20% ^15^N_2_ gas injected and for the two nitrogen atoms and the two hours of ^15^N_2_ incubation. These shoot, root, and nodule values were added together to give the final μg N_2_ fixed/h/plant. The Atom % values for both the shoot and root fractions in the water controls were 0.368, matching the ^15^N_2_ natural abundance and confirming that the assay worked well.

### Bacteroid isolation and protein measurement

Harvested nodules from plants inoculated with A34 and 4242 derivative strains labelled with constitutively sYFP (OPS1875 and OPS1877, respectively), were crushed with a pestle in a mortar with 2 ml of mulching buffer (100 mm potassium phosphate buffer pH 7.4, 300 mm sucrose, 2 mm MgCl_2_). Debris was filtered through two layers of gauze. The filtrate was loaded onto a 30 ml preformed Percoll gradient (43% Percoll, 57% Percoll buffer consisting of 232.4 mm potassium phosphate buffer pH 7.4, 697 mm sucrose, 4.65 mm MgCl_2_, centrifuged at 35,000 × *g*, 30 min, 4 °C in a swing-out rotor) and centrifuged at 35,000 × *g*, 4 °C for 20 min. To estimate bacteroid densities, identical gradients were loaded with colored beads of defined densities (Cospheric LLC, Somis, CA, USA). Bacteroid fractions were aspirated and loaded on a fresh Percoll gradient and processed as above. Aspirated pure bacteroid fractions were washed twice in resuspension buffer (50 mm potassium phosphate buffer pH 7.4, 2 mm MgCl_2_) before further use. Bacteroids were subsequently lysed using BugBuster (Sigma Aldrich) according to the manufacturer's recommendations, and protein was measured using the Bradford assay (Sigma Aldrich).

### Flow cytometry analysis

An Amnis Cellstream (Luminex ltd.) with autosampler and equipped with a 488 nm laser to excite sYPF2 was used for flow cytometer. Flow rates were set to low speed/high sensitivity (3.66 *µ*l/min) and the denser population in forward scatter-side scatter (FSC-SSC) was defined as “nodule.” Pea and bean bacteroid samples (sYFP2 labeled with Tn7 to enable detection, using strains OPS1875 and OPS1877 for A34 and 4242 derivatives, respectively. A total of 20,000 events in the nodule population were counted for each sample. Using Cellstream Analysis 1.3.384 software, the nodule population was afterward gated based on FSC (threshold >0) and the aspect ratio of SSC (threshold >0.4) defining the “singlets” population. Then, singlets events were gated based on their fluorescence emission of sYFP2 emission was detected at 528/46 and the events above 3,000 fluorescent units (FI) units were defined as the “yellow” population. Three technical replicates of six independent bacteroid isolations were processed and the number of events·mL^−1^ recorded.

### Confocal imaging

Five independent free-living cultures with cells expressing constitutive mCherry on pOPS0875 (strains OPS3405 and OPS3411, derivatives of A34 and 4242, respectively) were grown in UMS with 0.1% agarose, so they could be fixed for confocal imaging, and normalized to the same OD_600nm_. Nodules were placed in a molten 8% w/v solution of agarose. After the agar solidified, cubes with one nodule were sectioned longitudinally to 100 *μ*m thickness using a Leica VT1200S vibratome. Nodule sections were imaged with a Zeiss LSM 880 Airy Scan confocal microscope and the ZEN Black software. mCherry was visualized using a 561 nm laser and emission detected between 598 and 649 nm, sfGFP was visualized using a 488 nm wavelength laser and emission detected between 498 and 562 nm.

### Image analysis and volume calculations

Fluorescent nodules were analyzed using Fiji/ImageJ 2.9.0/1.53t. To establish the total infected area for each nodule, we used the NifA-dependent sfGFP signal (green channel). To enable comparable thresholding, we processed each image as follows: to subtract background noise, first, we subtracted 20 from each pixel intensity and then subtracted background using a sliding paraboloid of a 200-pixel radius. Then, we enhanced local contrast (CLAHE; blocksize 500, histogram bins 256, max. slope 6.00) to account for uneven signal intensity. Lastly, we used a median filter (3.00 px) to smooth edges before thresholding (otsu with manual correction where needed) the infected area. To determine the infected zone (i.e. the inner part of each nodule, excluding epidermis and cortex, where infected cells are found), we used the above determined infected areas to manually establish the borders. For the whole nodule area, the white light channel was used with inverse thresholding and manual correction as needed.

For bean nodules, the following parameters were obtained: the total area of each nodule section (A_nod_) and both maximum and minimum Feret's diameter (F_dmax_ and F_dmin_); the total infected area as determined by green-fluorescent areas containing *sfGFP*-expressing bacteroids (A_i_); the area of the infected zone (A_iz_) and both maximum and minimum Feret's diameter. Being approximately spherical, we calculated the volumes of both the whole bean nodule and the infected zone as follows:

$$V=\frac{4}{3}AF_{d}$$

Both maximum and minimum volumes (using F_dmax_ and F_dmin_) were calculated and the mean volume was taken as our final volume. The fractional colonization density (f_c_) of infection in the infected zone was calculated using the ratio A_i_/A_iz_ which was then used to calculate the total infected volume per nodule.

For pea nodules, we assumed that both the infection zone and the nodule as a whole took a cylindrical form, with an uninfected spherical cap at the distal end of the nodule. The infected area, infection zone, and total nodule area were determined as described for bean nodules. The cylindrical body of the nodule and the cap were then dealt with separately. For the cap, we treated the image as a 2D segment of the spherical cap. The chord length (c) and the sagitta (h) of the circular segment were measured. The volume of the uninfected spherical cap (V_c_) was calculated as follows:

$$V_{c}=\frac{1}{6}\pi h(3c^{2}+h^{2})$$

For the cylindrical body of the nodule and the infected zone within we calculated the length of the cylinders (l_c_) by using the skeletonize function of Fiji to create a midline of the nodule and then measuring this from end to end. Using the determined areas (in case of the whole nodule body excluding the cap area) the average width (d) of each was calculated (d = A/l_c_). The volumes of both the nodule body cylinder (V_b_) and the infected zone (V_iz_) were calculated then as follows:

$$V=\frac{Ad\pi }{4}$$

Total nodule volume (V_n_) was then calculated $\left(V_{n}=V_{c}+V_{b}\right)$. The total infected volume (V_i_) was again calculated using the ratio of infected tissue within the infected zone $\;(V_{i}=V_{iz}*A_{i}/A_{iz})$.

### Serial block face scanning electron microscopy (SBF-SEM) imaging and reconstruction

Selected nodules were immersed in 8% w/v agar, and these agar blocks containing fixed and embedded nodules were mounted on aluminum pins using conductive epoxy resin, sputter coated with gold and imaged on a Zeiss Merlin VP compact coupled with a 3view2XP chamber and an OnPoint backscattered electron detector (both from Gatan/Ametek, Pleasanton, CA). Samples were imaged by SBF-SEM, using the following imaging conditions: 1.8 kV accelerating voltage, 100% focal charge compensation, 20 *μ*m aperture, 7 nm pixel size, 3 to 4 μs pixel time, 100 nm section thickness. Data were assembled into 3D volumes and aligned using ETomo (part of the IMOD software package, v4.12.47; (Kremer et al. 1996)), and 3D reconstruction was performed using 3dmod (also part of IMOD). Bacteroids were segmented manually by tracing structures in each slice, and surface meshing and capping were performed in 3dmod. For packing data, individual volume datasets were trimmed from main data sets at defined volumes (X, 8 *μ*m, Y 8 *μ*m by Z 2 *μ*m). Within these individual packing datasets, every bacteroid was segmented to determine the percentage of plant cell volume occupied by bacteroids. Graphs were generated in Graphpad Prism (v10.0.1) and the figure generated using Inkscape (v1.0.2).

### Quantitative proteomic analysis

Bacteroids were obtained from nodules of bean and pea plants inoculated with Rlp4292 and RlvA34, respectively, as described above. Bacteroids were isolated from nodules of three plants each of bean and pea and processed as three independent replicates. Bacteroid soluble proteins were extracted as described (Schulte et al. 2021), precipitated in acetone in the presence of NaCl and the resulting pellets dissolved in 50 *µ*l of 8 m urea/0.1 m Tris (pH 8). A 5 *µ*l aliquot was used to quantify protein by Bradford assay. The remainder of all samples was adjusted to 4.6 mm dithiothreitol (DTT) by adding 0.9 *µ*l of 235 mm DTT, and the samples were incubated at 37 °C for 30 min. Then, 0.8 *µ*l of 545 mm iodoacetamide was added to final concentration of 9 mm and samples incubated for 30 min at room temperature. Two µl (1 *µ*g) of trypsin/Lys-C mix (Promega) was added and the samples incubated at 37 °C for 5 h, diluted one in six with 0.1 m Tris (pH 8) and digested at 37 °C for a further 16 h. The reaction was stopped by adding 20% (v/v) trifluoracetic acid to a final concentration of 1%.

For loading to the liquid chromatography mass spectrometry analysis, aliquots were prepared from each sample containing 380 ng of digested bacteroid protein. Each aliquot was spiked with 12.5 fmole Hi3 Phos B standard (Waters, 186006011) for absolute quantification. Each biological replicate was run three times on a nanoLCMS system comprising an LTQ-Orbitrap (Thermo Fisher Scientific, Hemel-Hempstead, UK) and a nanoACQUITY UPLC system (Waters, Manchester, UK as described previously (Schulte et al. 2021) except a longer gradient was used from 3% to 40% acetonitrile over 100 min.

All raw files were processed individually in MaxQuant (1.5.0.0) with protein sequence databases for RlvA34 and Rlp4292 (Green et al. 2019). The PHS2_RABIT (P00489) sequence, from which the Hi3 standard peptides are derived, was added to the databases, along with the MaxQuant contaminants database. MaxQuant parameters were set to: trypsin as enzyme with no missed cleavages; precursor and fragment tolerances 6 ppm/0.5 Da; carbamidomethyl on C as fixed; and oxidation on M and acetylation of protein N-terminus as variable modification. The MaxQuant output tables modificationSpecificPeptids.txt were merged for RlvA34 and Rlp4292 and used to calculate absolute protein abundances using the top3 approach and the Hi3 standard (see above). An in-house script (top3) was applied to the merged modificationSpecificPeptids.txt table, which calculates the average of the top three unique peptides for each protein. No missed cleavage was allowed, and only unmodified peptides and peptides with oxidized methionine were used. If the same peptide was found unmodified and oxidized, the sum of both was used. Data were normalized using the RegrRun procedure (Kultima et al. 2009). The mass spectrometry proteomics data have been deposited to the ProteomeXchange Consortium via the PRIDE (Perez-Riverol et al. 2022) partner repository with the dataset identifier PXD058609.

To calculate the absolute abundance of each protein as fmole in the loaded sample, each protein abundance was divided by the Hi3 standard abundance and multiplied by 12.5 resulting in the protein abundance value (A_b_). The results were transformed into protein copy numbers (C_p_) based on the Avogadro constant (N_A_) using 6.0221413×10^8^ copies per fmole. Each 380 ng loaded sample of bacteroid protein was calculated to have been derived (B_D_) from 1,202,531 bean and 608,974 pea bacteroids, based on protein contents of 0.32 pg protein/bacteroid for bean and 0.62 pg protein/bacteroid for pea (Table 1). Copies per bacteroid where subsequently determined with the formula:

$$C_{p}\;(bacteroid)\;=\frac{C_{p}}{B_{D}}$$

Copies per bacteroid volume were calculated by:

$$C_{p}\;(bacteroid\;vol)\;=\frac{C_{P}\;(bacteroid)}{B_{vol}}$$

where B_vol_ = bacteroid volumes of 0.93 and 4.50 *µ*m^3^/bacteroid for bean and pea (Table 1). Copies per nodule were calculated by:

$$C_{p}\;(nodule)\;=\frac{C_{P}\;(bacteroid)}{B_{nod}}$$

where B_nod_ = bacteroids per nodule of 5.28×10^8^ and 1.63×10^8^ bacteroids for bean and pea (Table 1). Copies per nodule volume were calculated by:

$$C_{p}\;(nodule\;volume)\;=\frac{C_{P}\;(nodule)}{Nod_{vol}}$$

where Nod_vol_ = Mean nodule volume of 6.07 and 4.61 mm^3^/nodule for bean and pea (Table 1).

Copies per unit protein were calculated by:

$$C_{p}\;(unit\;protein)\;=\frac{C_{P}\;(bacteroid)}{B_{P}}$$

where B_P_ = bacteroid protein content of 0.32 and 0.62 pg/bacteroid for bean and pea (Table 1). Welch's two-sample T test was used to identify proteins common to both RlvA34 and Rlp4292 bacteroids whose expression was significantly different between each bacteroid type (*P* < 0.05). The mass of each protein detected in Rlp4292 and RlvA34 bacteroids was calculated and used to rank proteins in RlvA34 and Rlp4292 bacteroids and compare the percentage proteome by mass to proteins ranked from most to least abundant.

RNA-Seq data from bacteroid of *R. leguminosarum bv. phaseoli* 4292 and *R. leguminosarum* bv. viciae A34, isolated from bean and pea plants, respectively (Green et al. 2019), were used to calculated normalized counts using the EDGE-pro (Magoc et al. 2013) and DESeq2 (Love et al. 2014) software packages. Only genes present in the proteome were further analyzed. Pearson correlation between proteome and transcriptome for bean and pea bacteroid was performed using Prism (version 10.4.0).

### Accession numbers

Sequence data from this article can be found in the GenBank data library under accession numbers NZ_AQZR00000000.1 for *R. leguminosarum* bv. phaseoli 4292, and ARRT00000000.1 for the symbiotic plasmid of pRLJI1 in *R. leguminosarum* bv. viceae 248, with locus tags for protein sequence database used in this study listed in Green et al. (2019), Supplementary Table S4.

## Supplementary Material

kiaf613_Supplementary_Data

## Data Availability

Proteomic data are available via ProteomeXchange with identifier PXD058609. Flow cytometer data is available at http://flowrepository.org, experiment codes are FR-FCM-Z623 and FR-FCM_Z624 for pea and bean bacteroids respectively.

## Supplementary data

The following materials are available in the online version of this article.

**Supplementary Figure S1**. Density gradient centrifugation of bean and pea bacteroids.

**Supplementary Table S1**. Rates of N_2_-fixation of different strains of pea and bean nodulating rhizobia. Supplementary Video 1 of pea and bean nodules acquired by serial block face scanning electron microscopy showing segmented bacteroids.

## Dive Curated Terms

The following phenotypic, genotypic, and functional terms are of significance to the work described in this paper:

ATP CHEBI: CHEBI:15422

acetylene CHEBI: CHEBI:27518

NAD CHEBI: CHEBI:13389
