# Supplementary material for: Developmental fates and N2-fixing efficiency of terminally-differentiated versus undifferentiated bacteroids from legume nodules
Source: Plant Physiol. 2025 Dec 9;200(3):kiaf613. doi: 10.1093/plphys/kiaf613 (PMC13098155; doi:10.1093/plphys/kiaf613)
Supplement: kiaf613_Supplementary_Data [file kiaf613_Supplementary_Data.zip › Supplementary data.docx]

**SUPPLEMENTARY DATA**

Developmental fates and N_2_-fixing efficiency of terminally-differentiated versus undifferentiated bacteroids from legume nodules

**A.**

|  | Pea (cv Avola) | | Bean (cv Tendergreen) | |
| --- | --- | --- | --- | --- |
| **C_2_H_2_ Reduction rate** | RlvA34 (n=15) | Rlv3841 (n=14) | Rlp4292 (n=10) | RlpCE3 (n=5) |
| Mean fixation  (µmol C_2_H_4_ h^−1^ g^−1^) | 13.8965 | 14.39194 | 6.621172 | 8.541808 |
| STDEV | 2.043698 | 1.791497 | 1.645092 | 1.91326 |
| SEM | 0.527681 | 0.478798 | 0.520224 | 0.855636 |
| *p*-value | 0.4948 | | 0.064 | |

**B.**

| **C_2_H_2_ Reduction rate** | Rlv3841 + Avola (n=4) | Rlv3841 + Frisson (n=5) |
| --- | --- | --- |
| Mean fixation  (µmol C2H4 h−1 g−1) | 11.34725 | 8.340559 |
| STDEV | 0.528092 | 1.848752 |
| SEM | 0.264046 | 0.826787 |
| p-value | 0.0170 | |

**C.**

| **^15^N_2_ Fixation rate** | RlvA34 Avola (n=6) | Rlv3841 Avola (n=10) |
| --- | --- | --- |
| Mean fixation  (µmol 15N h−1 µg−1) | 2.349 | 2.930 |
| STDEV | 0.771 | 0.99 |
| SEM | 0.244 | 0.41 |
| p-value | 0.2104 | |

**Table S1.** **Rates of N_2_-fixation of different strains of pea and bean nodulating rhizobia.** (A) Rates of acetylene reduction per g fresh weight of nodule of two pea (RlvA34 and Rlv3841) and bean (Rlp4292 and RlpCE3) nodulating rhizobia. (B) Rates of acetylene reduction per g fresh weight of nodule of strain Rlv3841 on two cultivars of pea. The parent of strain Rlp4292 (strain 8002) has also been compared against multiple bean nodulating rhizobia and a different bean cultivar and shown to be highly effective (Mwenda et al., 2018). (C). Rates of ^15^N_2_ fixation per g fresh weight of nodule of two pea nodulating rhizobia.

Plants harvested at flowering (35 days post inoculation for beans and 28 days post inoculation for pea) at peak nitrogenase activity. Values represent the average with calculated standard deviation and standard error. Student’s unpaired T-Test, with significance set at *p*-value < 0.05.

**Mwenda GM, O'Hara GW, De Meyer SE, Howieson JG, Terpolilli JJ** (2018) Genetic diversity and symbiotic effectiveness of *Phaseolus vulgaris*-nodulating rhizobia in Kenya. Syst Appl Microbiol **41:** 291-299

**Figure S1.** **Density gradient centrifugation of bean and pea bacteroids**. Rlp4292 bean (left) and RlvA34 pea (right). Each bacteroid fraction from nodule crude extracts is indicated by red arrows. An identical density gradient (middle) was loaded with colored beads of different densities. Separated beads on the gradient are labelled with their nominal density in the same color as the beads.
